# Association between the triglyceride–glucose index and left ventricular myocardial work indices in patients with coronary artery disease

**DOI:** 10.3389/fendo.2024.1447984

**Published:** 2024-10-24

**Authors:** Xuyang Meng, Baoyu Feng, Chenguang Yang, Yi Li, Chenxi Xia, Ying Guo, Xiang Wang, Fang Wang

**Affiliations:** ^1^ Department of Cardiology, Beijing Hospital, National Center of Gerontology, Institute of Geriatric Medicine, Chinese Academy of Medical Sciences, Beijing, China; ^2^ Graduate School of Peking Union Medical College, Chinese Academy of Medical Sciences, Beijing, China; ^3^ Department of Clinical Trial Center, Beijing Tiantan Hospital, Capital Medical University, Beijing, China; ^4^ Fifth School of Clinical Medicine, Peking University, Beijing, China

**Keywords:** coronary artery disease, triglyceride-glucose index, myocardial work, cardiac function, GWW

## Abstract

**Background:**

Triglyceride–glucose (TyG) index, as an effective surrogate marker of insulin resistance, has shown predictive value in the risk of heart failure in patients with coronary artery disease (CAD). This study aims to investigate the correlation between TyG index and myocardial work measurements in CAD, and to explore its role in detecting early subclinical cardiac dysfunction.

**Methods:**

This cross-sectional study included 267 patients diagnosed with CAD and excluding left ventricular myocardial dysfunction in Beijing Hospital. Participants were divided into two groups according to the TyG index level, and myocardial work measurements were compared between groups. The correlation was explored between gradually increased TyG index and subclinical myocardial function in CAD patients.

**Results:**

We observed that TyG index was significantly correlated with the global waste work (GWW), and the value of GWW increased progressively with the elevation of TyG index. After adjusting for the effects of confounding factors, TyG index was still independently associated with GWW.

**Conclusion:**

An elevated TyG index was independently correlated with early subclinical myocardial dysfunction in CAD patients. Our study demonstrated that the strict control of TyG index may be conducive to forestall the progression of clinical heart failure in CAD patients.

## Introduction

Coronary artery disease (CAD) is one of the major causes of left ventricular systolic dysfunction, progressive decline in cardiac function may occur in patients with CAD even after revascularization, which subsequently leads to heart failure (HF) (1, 2). Meanwhile, due to the presence of cardiovascular risk factors, adverse myocardial remodeling and dysfunction appear much earlier than the onset of HF symptoms (3). Therefore, it is essential for clinicians to identify left ventricular dysfunction, early detect and control cardiovascular risk factors. Type 2 (T2) diabetes mellitus (DM) is a major risk factor for cardiovascular diseases, and doubles the risk of developing HF (4). Insulin resistance is a critical mechanism in developing T2DM and significantly correlated with adverse cardiovascular outcomes (5–8). Composed of fasting triglyceride (TG) and fasting blood glucose (FBG), the triglyceride–glucose (TyG) index has been confirmed as a surrogate marker of insulin resistance (9). Existing research has suggested that the TyG index may be a valuable indicator for assessing the risk of HF incidence in patients with CAD (6), but its clinical value in the subclinical stage of HF has not been confirmed by current studies.

Left ventricular ejection fraction (LVEF) is a commonly used parameter to evaluate left ventricular systolic function in clinical Settings, but its limitations are increasingly apparent in preclinical HF, and it cannot explain local myocardial damage (10). Strain imaging by noninvasive speckle-tracking echocardiography (STE) has become a valuable tool in the assessment of left ventricular function (11). Global longitudinal strain (GLS) has been shown to be superior to LVEF in the detection of early subclinical myocardial dysfunction, but both measures are afterload dependent (12, 13). Myocardial work (MW), which incorporates left ventricular pressure into the strain measurement, offsets the disadvantages of GLS alone for detecting LV dysfunction (14). The current study investigated the association between the TyG index level and left ventricular MW indices in patients with CAD, and to provide clinical evidence for the correlation between elevated TyG index and the myocardial remodeling during subclinical stage.

## Methods

### Study design and population

**Figure 1:**
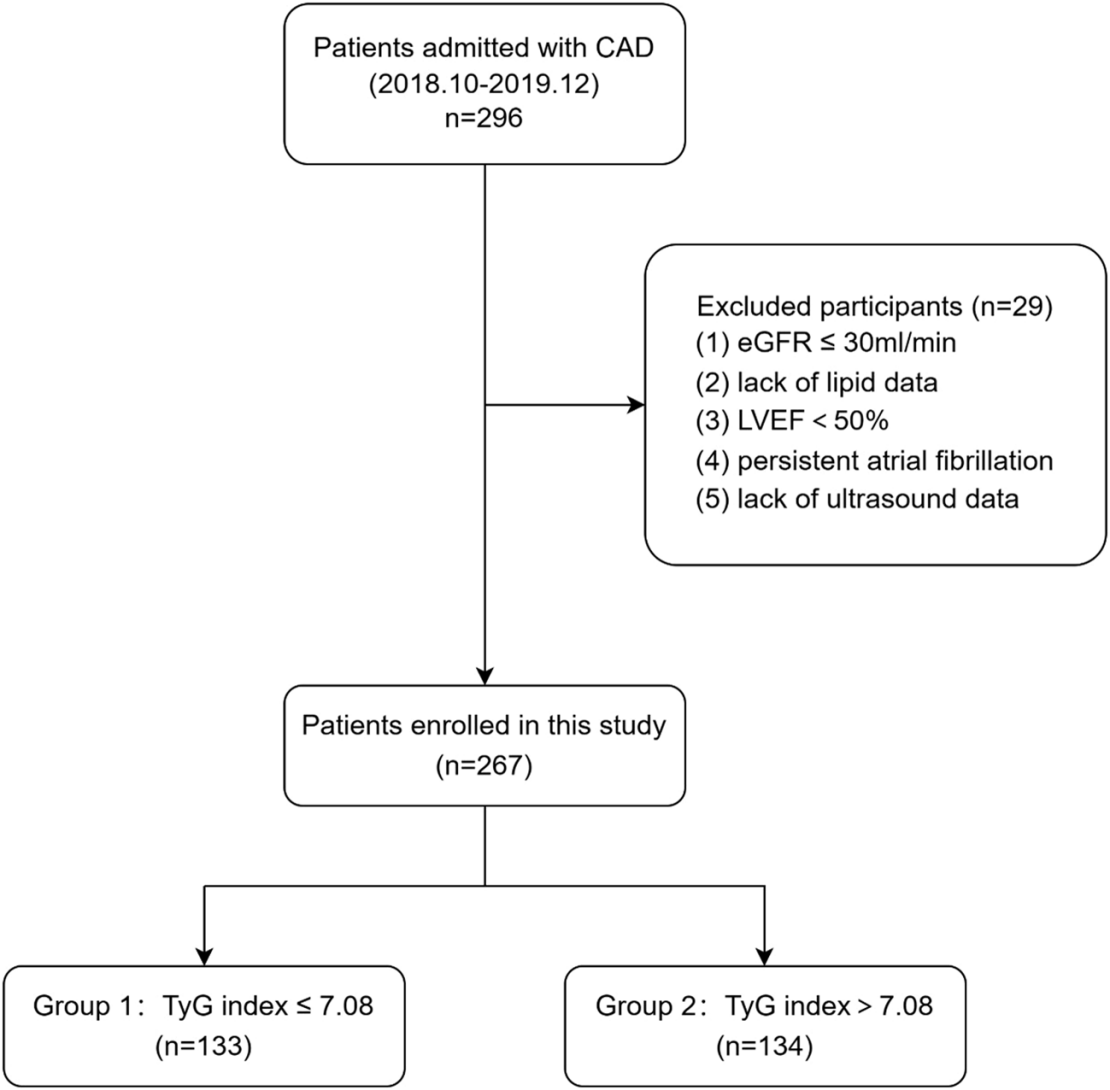
Flowchart of study patients. CAD, coronary artery disease; eGFR, estimated glomerular filtration rate; LVEF, left ventricular ejection fraction; TyG, triglyceride–glucose.

This cross-sectional study was performed in Beijing Hospital in China between October 2018 and December 2019. A total of 296 participants with stable CAD were enrolled during hospitalization. Stenosis ≥50% in at least one major coronary artery or its main branch was identified as CAD. After excluding patients with LVEF ≤ 50%, persistent atrial fibrillation, lack of lipid or ultrasound data and estimated glomerular filtration rate (eGFR) < 30 ml/min, we included 267 patients in this current analysis eventually. **Figure 1** shows the flowchart of this study. The eligible participants were divided into two groups according to the median TyG index: TyG index≤ 7.08 group (n = 133) and TyG index > 7.08 group (n = 134).

The study was accordance with the principles of the Declaration of Helsinki and was approved by the Ethics Committee of Beijing Hospital. Informed consent was obtained from all study participants.

### Measurements and definitions

We collected the sociodemographic characteristics (age, sex, height, weight, smoking status, and drinking status), medical history (diabetes mellitus, hypertension, and hyperlipidemia), antidiabetic drugs (oral hypoglycemic drugs and insulin) and laboratory test results of each participant from medical records from Beijing Hospital. Blood samples were obtained from all the participants after at least 8 hours of fasting for the measurement of FBG, creatinine, total cholesterol (TC), TG, low-density lipoprotein cholesterol (LDL-C), glycated hemoglobin A1c (HbA1c) and hemoglobin (Hb). Patient’s systolic blood pressure (SBP), diastolic blood pressure (DBP), and heart rate (HR) were recorded in a resting state just before echocardiography examination.

Body mass index (BMI) was calculated as weight (kg) divided by the squared height (m). Estimated glomerular filtration rate (eGFR) was calculated according to the Chronic Kidney Disease Epidemiology Collaboration creatinine equation (15). The TyG index was calculated as follows: Ln (fasting TG [mg/dL] × FBG [mg/dL]/2) (16).

The Glucose metabolism status was defined according to the American Diabetes Association (ADA) criteria (17). DM was diagnosed when patients had the following: an FBG level ≥7.0 mmol/L, 2-hour plasma glucose level ≥11.1 mmol/L according to the oral glucose tolerance test, HbA1c ≥6.5%, or diabetes history.

Since the patients’ cardiac structure and function changes were not caused by one coronary artery lesion alone, we calculated GENSINI scores based on coronary angiography to evaluate the severity of CAD (18). The degree of lesion of each vessel was quantitatively assessed: Luminal stenosis ≤ 25% was 1 point, 26% ~50% was 2 points, 51% ~75% was 4 points, 76% ~90% was 8 points, 91% ~99% was 16 points, and 100% was 32 points. The score was multiplied by the corresponding coefficient, and the final score of the lesion was the sum of the branching scores.

### Echocardiographic analysis

Before coronary angiography, echocardiography was performed using a Vivid E95 ultrasound system (GE Vingmed Ultrasound, Norway) with electrocardiogram connected. All images, consisting of three to five consecutive cardiac cycles triggered to the QRS complex, were stored in RAW format for offline analysis. Left atrial diameter (LAD) and left ventricular end diastolic diameter (LVEDD) were measured on the parasternal long-axis view. Left ventricular end diastolic volume (LVEDV) was calculated using the modified biplane Simpson method, with LVEF subsequently determined.

### GLS and MW analysis

Two-dimensional grayscale images from the apical two-, three-, and four-chamber views were acquired to enable GLS analysis by STE. GLS was quantified using automated functional imaging from a dedicated workstation (EchoPAC version 204). These outlines can also be adjusted manually to conform to the myocardium. The increase or decrease in strain was expressed as absolute values.

MW indices were obtained using a pressure-strain loop (PSL) area module that was constructed using left ventricular pressure values on the vertical axis and strain on the horizontal axis. Peak left ventricular systolic pressure is estimated to be noninvasive systolic cuff pressure. Once SBP was inputted into the software, the following parameters were generated:

1. Global work index (GWI): Total work within the area of the LV-PSL from mitral valve closure to mitral valve opening.
2. Global constructive work (GCW): The work performed during myocardial shortening in systole and lengthening in isovolumic diastole.
3. Global waste work (GWW): The work performed by myocytes during myocardial elongation in systole and shortening in isovolumic diastole.
4. Global work efficiency (GWE): GCW/(GCW + GWW).

### Statistical analysis

Continuous variables conforming to normal distribution by Kolmogorov-Smirnov test were expressed as mean ± standard deviation, and differences between two groups were compared by t-test. Data with skewed distributions were expressed as median (interquartile range) and comparisons between two groups using Mann-Whitney U test. Categorical variables were expressed as numbers(n) and percentages (%), and chi-square test was used for comparison between groups. Pearson’s or Spearman’s correlation analyses were used to assess associations between left ventricular GLS and MW variables and potential cardiac risk factors.

We assessed the association between the TyG index and the risk of GLS and MW variables using a multivariate linear regression model and restricted cubic spline (RCS). We used three models to adjust for potential confounders: Model 1 was unadjusted; Model 2 was adjusted for age, gender, smoking and drinking; Model 3 was further adjusted for SBP, BMI, HbA1c, TC and LDL-c. In these models, the average value of the TyG-index quartiles was used as the continuous variable in the regression model to conduct linear trend tests. Response Surface Methodology was used to describe the relationship between TG, FBG and GWW.

In the subgroup analysis, we examined the relationship between GWW and the TyG index according to gender (male vs. female), age (<65 vs. ≥65 years), eGFR (≥90 vs. <90 ml/min), hypertension (yes vs. no), and glucose metabolism states (DM vs. Non-DM). The product of grouping variables and intervening variables was put into the model to evaluate the interaction effects.

All statistical analyses were performed using SPSS, R and Graphpad Prism software. All P values were 2-sided, and statistical significance was defined as P < 0.05.

## Results

### Baseline characteristics

A total of 296 patients with coronary artery disease were included in this study, and 29 patients were excluded according the inclusion and exclusion criteria. Finally, a total of 267 patients were included in the study. **Figure 1** shows the flowchart of the patient enrollment.

The mean age of the 267 participants was 64.3 ± 9.8 years, 133 of them with TyG index ≤ 7.08 were classified as Group 1, 134 with TyG index > 7.08 were classified as Group 2. **Table 1** shows the baseline characteristics. Participants in Group 1 had a higher age and HDL-c level (both P<0.05), but their DBP, LDL-c, TC, TG, FBG, HbA1c and E/A ratio were lower than those in Group 2 (all P<0.05). The other routine echocardiographic parameters showed no statistically significant differences between the two groups. In Group 2, the GLS was observed significantly impaired (P=0.001), while the increase of GWW (P<0.001) and the decrease of GWE (P=0.001) were both statistically significant.

**Table 1:** Baseline characteristics of participants.

Variables	Total (n=267)	Group1 (n=133)	Group2 (n=134)	*P* value
Age (years)	64.3 ± 9.8	65.6 ± 9.7	62.9 ± 9.7	0.022
Male (n, %)	200 (75.7%)	102 (76.7%)	98 (73.1%)	0.502
Smoking (n, %)	143 (53.6%)	65 (48.9%)	78 (58.2%)	0.126
Drinking (n, %)	119 (44.6%)	52 (39.1%)	67 (50.0%)	0.073
BMI (kg/m2)	26.0 (24.1, 27.5)	25.7 ± 2.9	26.3 (24.1, 27.7)	0.363
SBP (mmHg)	134.3 ± 17.1	132.4 ± 16.4	136.1 ± 17.7	0.079
DBP (mmHg)	76.0 ± 10.4	73.6 ± 9.7	78.4 ± 10.7	<0.001
eGFR (ml/min)	91.7 (75.4, 111.6)	92.8 ± 26.8	93.1 (77.9, 115.3)	0.184
TC (mmol/L)	3.7 (3.2, 4.2)	3.5 ± 0.8	3.9 ± 0.9	<0.001
TG (mmol/L)	1.3 (0.9, 1.8)	0.9 ± 0.3	1.7 (1.5, 2.2)	<0.001
HDL-C (mmol/L)	1.0 (0.9, 1.2)	1,1 (0.9, 1.3)	0.9 (0.8, 1.1)	<0.001
LDL-C (mmol/L)	2.2 (1.8, 2.7)	2.0 (1.7, 2.6)	2.4 (1.9, 2.8)	0.005
FBG (mmol/L)	5.6 (5.0, 6.8)	5.2 (4.8, 5.8)	6.6 (5.5, 7.8)	<0.001
HbA1c (%)	6.4 (5.9, 7.1)	6.1 (5.7, 6.7)	6.7 (6.2, 7.6)	<0.001
TyG index	7.1 ± 0.6	6.7 (6.4, 6.9)	7.5 (7.3, 7.8)	<0.001
GENSINI score	24.0 (16.5, 41.0)	24.0 (16.0, 40.0)	25.0 (17.0, 43.9)	0.574
Number of coronary arteries with ≥50% stenosis				0.066
1	61 (22.9%)	34 (25.6%)	27 (20.1%)	
2	101 (37.8%)	56 (42.1%)	45 (33.6%)	
3	105 (39.3%)	43 (32.3%)	62 (46.3%)	
Diabetes mellitus (n, %)	140 (52.4%)	51 (38.3%)	89 (66.4%)	<0.001
Hypertension (n, %)	188 (70.4%)	87 (65.4%)	101 (75.4%)	0.075
Hyperlipidemia (n, %)	209 (78.3%)	104 (77.4%)	105 (78.4%)	0.974
PCI history (n, %)	72 (27.0%)	36 (27.1%)	36 (26.9%)	0.970
Antidiabetic drugs				
oral hypoglycemic drugs (n, %)	105 (39.3%)	36 (27.1%)	69 (51.5%)	<0.001
Insulin (n, %)	34 (12.7%)	6 (4.5%)	28 (20.9%)	<0.001
Beta-blocker	187 (70.0%)	90 (67.7%)	97 (72.4%)	0.400
ACEI/ARB	148 (55.4%)	60 (45.1%)	88 (65.7%)	0.001
CCB	85 (31.8%)	40 (30.1%)	45 (33.6%)	0.036
anti-hyperlipidemic agents	266 (99.6%)	133 (100.0%)	133 (99.3%)	1.000
Echocardiographic characteristics				
LAD (mm)	35.0 (32.0, 37.0)	35.0 (32.0, 37.0)	35.0 (32.0, 37.0)	0.817
LVEDD (mm)	46.0 (43.0, 49.0)	45.9 ± 4.2	46.0 (44.0, 48.0)	0.839
LVEDV (ml)	97.0 (83.0, 113.0)	97.0 (83.0, 113.0)	97.0 (88.0, 108.0)	0.742
LVEF (%)	65.0 (60.0, 65.0)	65.0 (62.0, 65.0)	65.0 (60.0, 65.0)	0.063
E/A ratio	0.8 (0.7, 1.0)	0.9 (0.7, 1.0)	0.8 (0.6, 1.0)	0.018
GLS (%)	17.0 (15.0, 18.0)	17.0 (15.0, 18.5)	16.0 (14.0, 18.0)	0.001
GWI (mmHg%)	1838.8 ± 382.1	1869.5 ± 394.9	1808.3 ± 368.0	0.192
GCW (mmHg%)	2028.2 ± 427.5	2048.0 ± 432.9	2008.7 ± 422.8	0.454
GWW (mmHg%)	103.0 (73.0, 165.0)	92.0 (58.5, 153.0)	118.5 (83.0, 183.0)	<0.001
GWE (%)	0.94 (0.90, 0.96)	0.95 (0.92, 0.96)	0.93 (0.89, 0.95)	0.001

SBP, systolic blood pressure; DBP, diastolic blood pressure; BMI, body mass index; Hb, hemoglobin; eGFR, estimated glomerular filtration rate; HDL-C, high-density lipoprotein cholesterol; LDL-C, low-density lipoprotein cholesterol; TC, total cholesterol; TG, triglyceride; FBG, fasting blood glucose; HbA1c, glycated hemoglobin A1c; TyG, triglyceride–glucose; PCI, percutaneous coronary intervention; ACEI, angiotensin converting enzyme inhibitor; ARB, angiotensin receptor blocker; CCB, calcium channel blockers; LAD, center atrial diameter; LVEDD, center ventricular end diastolic diameter; LVEDV, center ventricular end-diastolic volume; LVEF, center ventricular ejection fraction; GLS, global longitudinal strain; GWI, global work index; GCW, global useful work; GWW, global wasteful work; GWE, global work efficiency.

### Correlation analysis

**Figure 2:**
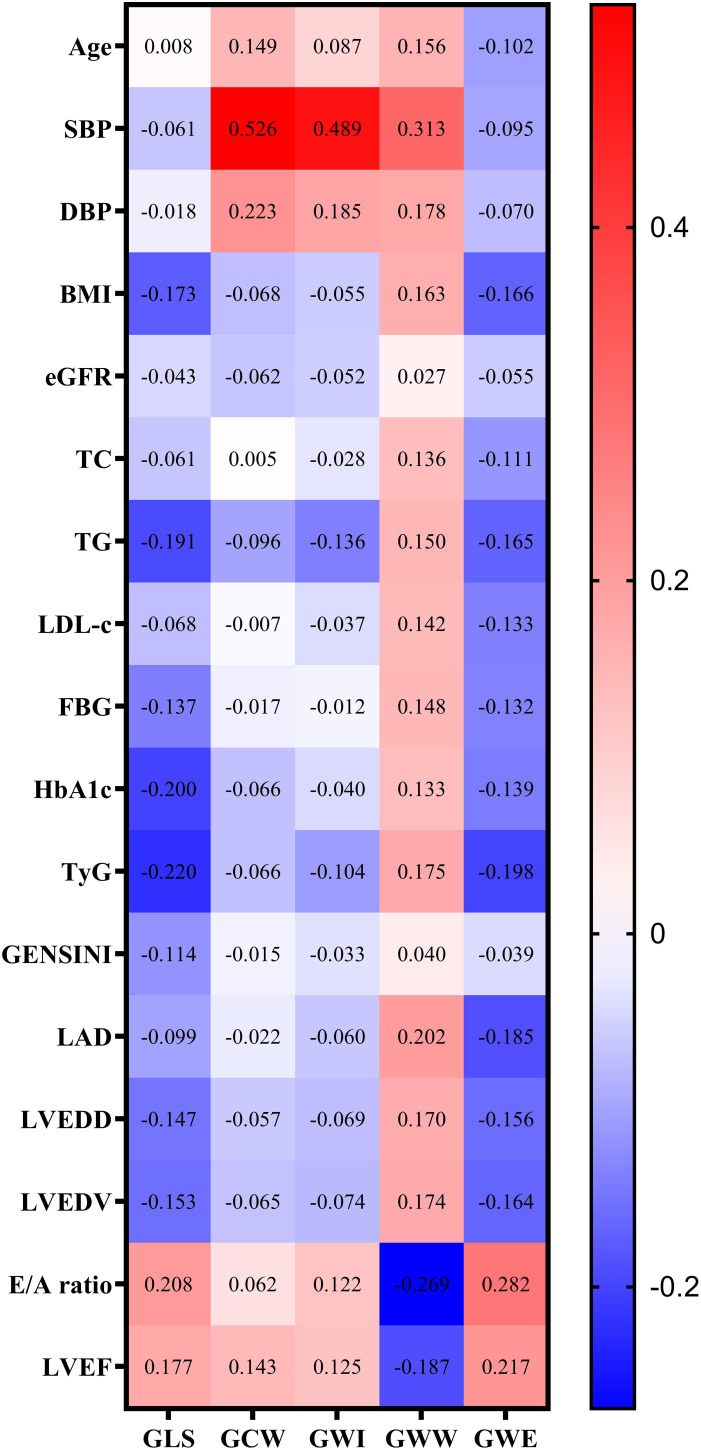
Correlation analysis of potential risk factors for GLS and MV parameters. Note: GWW was converted by natural logarithmic function. GLS, global longitudinal strain; MW, myocardial work; SBP, systolic blood pressure; DBP, diastolic blood pressure; BMI, body mass index; eGFR, estimated glomerular filtration rate; TC, total cholesterol; TG, triglyceride; LDL-C, low-density lipoprotein cholesterol; FBG, fasting blood glucose; HbA1c, glycated hemoglobin A1c; TyG, triglyceride–glucose; LAD, left atrial diameter; LVEDD, left ventricular end diastolic diameter; LVEDV, left ventricular end-diastolic volume; LVEF, left ventricular ejection fraction; GWI, global work index; GCW, global useful work; GWW, global wasteful work; GWE, global work efficiency.

**Figure 2** shows the correlations between potential risk factors and GLS and MW parameters. Correlation analysis showed that age, SBP, DBP, BMI, TC, TG, LDL-c, FBG, HbA1c, TyG index, LAD, LVEDD, LVEDV, E/A ratio and LVEF were associated with GWW in CAD patients. TyG index was positively correlated with GWW (P=0.004), and negatively correlated with GLS (P<0.001) and GWE (P=0.001).

Multivariable linear regression models were constructed to further investigate the independent correlation between TyG index and GWW in CAD patients (**Table 2**). TyG index was still correlated with GWW after adjusting for confounding factors in model 2 (β=0.160, 95% CI 0.041-0.279, P=0.008) and model 3 (β=0.152, 95% CI 0.016-0.287, P=0.029), indicating that elevated TyG index level may be independently associated with early left ventricular function impairment.

**Table 2:** Multivariate analysis of parameters associated between TyG index and GWW.

Model	β	95% CI	*P* value
Model 1	0.172	0.055-0.290	0.004
Model 2	0.160	0.041-0.279	0.008
Model 3	0.152	0.016-0.287	0.029

GWW was converted by natural logarithmic function.

Model 1: unadjusted.

Model 2: adjusted for age, sex, smoking and drinking.

Model 3: adjusted for Model 2 covariates + SBP, BMI, HbA1c, TC and LDL-C.

TyG, triglyceride–glucose; GWW, global wasteful work; CI, confidence interval; SBP, systolic blood pressure; BMI, body mass index; HbA1c, glycated hemoglobin A1c; TC, total cholesterol; LDL-C, low-density lipoprotein cholesterol.

**Figure 3:**
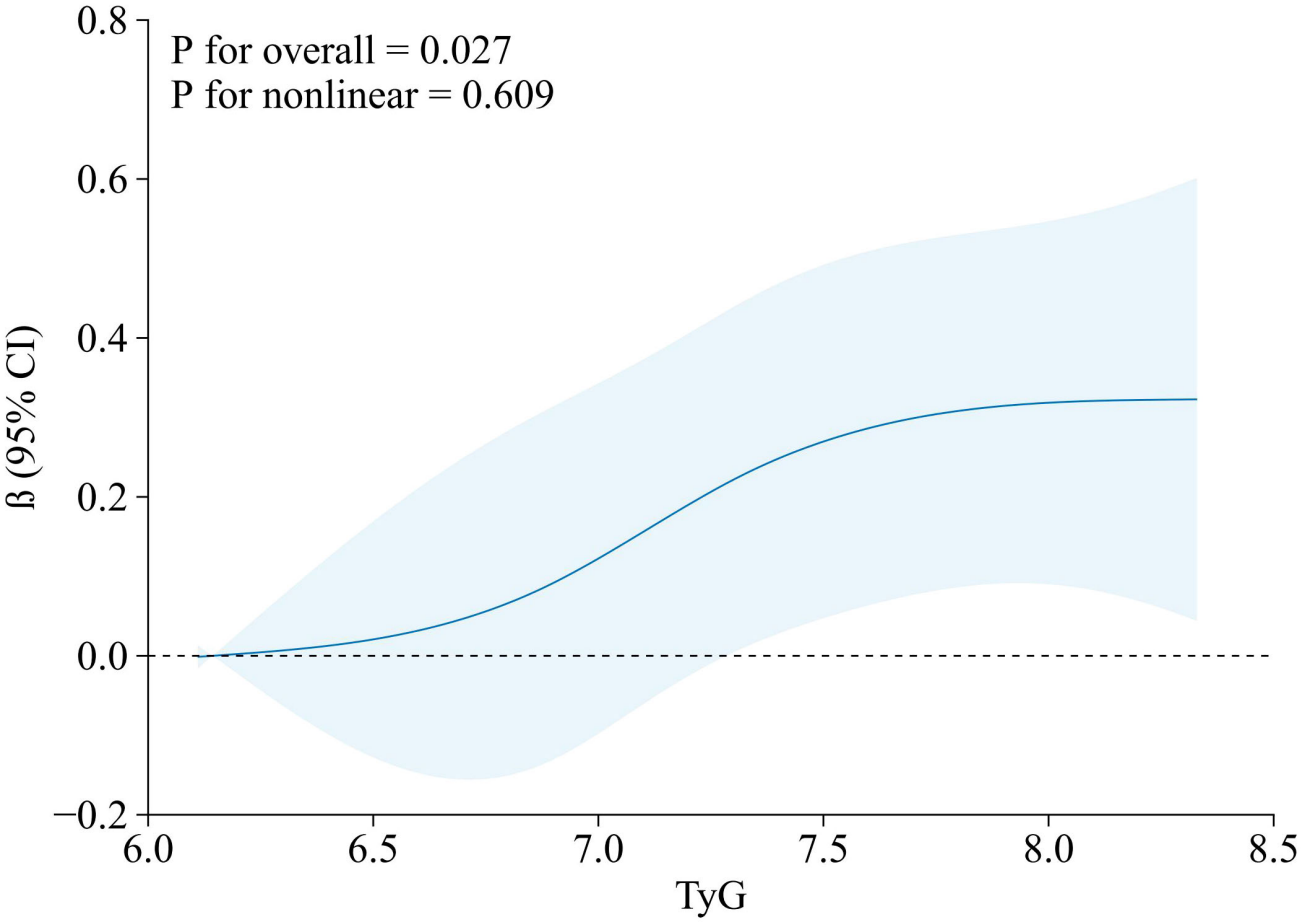
RCS model of the association between TyG index and GWW. Note: GWW was converted by natural logarithmic function. Data were fitted by a linear regression model, and the model was conducted with 4 knots at the 5th, 35th, 65th, 95th percentiles of TyG (reference is the 5th percentile). Solid lines indicate β, and shadow shape indicate 95% CIs. CI, confidence interval. RCS, restricted cubic spline; TyG, triglyceride–glucose; GWW, global wasteful work.

The results of the RCS are presented in **Figure 3**, which showed that TyG index was positively correlated with GWW.

**Figure 4:**
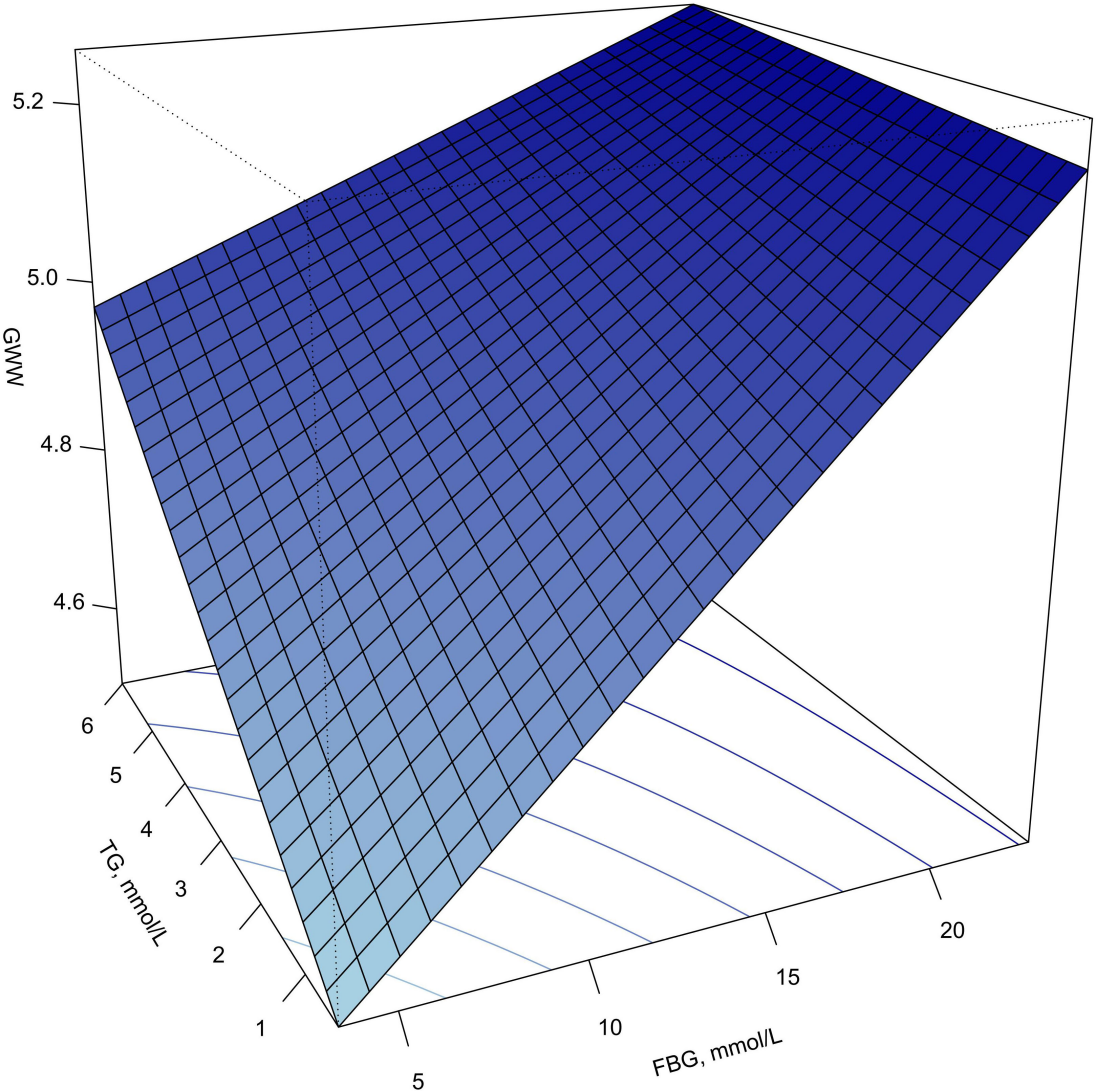
Response surface plot of the association of TG, FBG and GWW. Note: GWW was converted by natural logarithmic function. GWW, global wasteful work; TG, triglyceride; FBG, fasting blood glucose.

**Figure 4** shows the response surface plot of the association of TG, FBG and GWW. With the elevation of TG and FBG levels, the value of GWW showed an upward trend, indicating that the increasing of the wasted energy.

TyG index was divided into quartiles and included as variables in the regression model, and set the least quartile group as the reference group. **Table 3** presents the results of the trend test between TyG index and GWW. Higher value of TyG groups (Q3 and Q4 groups) were correlated with increased GWW (β=0.203; 95% CI 0.075-0.330; P=0.002) in model 1. After adjusting for age, sex, smoking, drinking, SBP, BMI, HbA1c, TC and LDL-c, the above conclusion still held (β=0.183; 95% CI 0.042-0.324; P=0.011).

**Table 3:** Trend test of changes in TyG index and GWW.

Model	Quartile of TyG index				P for trend
	Q1(<6.70) OR (95% CI)	Q2(6.70-7.08) OR (95% CI)	Q3(7.09-7.53) OR (95% CI)	Q4(>7.53) OR (95% CI)	
Model 1	Reference	1.058 (-0.152~0.264)	1.346 (0.090~0.503)	1.327 (0.076~0.490)	0.002
Model 2	Reference	1.066 (-0.139~0.267)	1.369 (0.113~0.516)	1.322 (0.070~0.488)	0.002
Model 3	Reference	0.970 (-0.231~0.171)	1.254 (0.027~0.425)	1.264 (0.005~0.463)	0.011

GWW was converted by natural logarithmic function.

Model 1: unadjusted.

Model 2: adjusted for age, sex, smoking and drinking.

Model 3: adjusted for Model 2 covariates + SBP, BMI, HbA1c, TC and LDL-C.

TyG, triglyceride–glucose; GWW, global wasteful work; CI, confidence interval; SBP, systolic blood pressure; BMI, body mass index; HbA1c, glycated hemoglobin A1c; TC, total cholesterol; LDL-C, low-density lipoprotein cholesterol.

### Subgroup analyses

**Figure 5:**
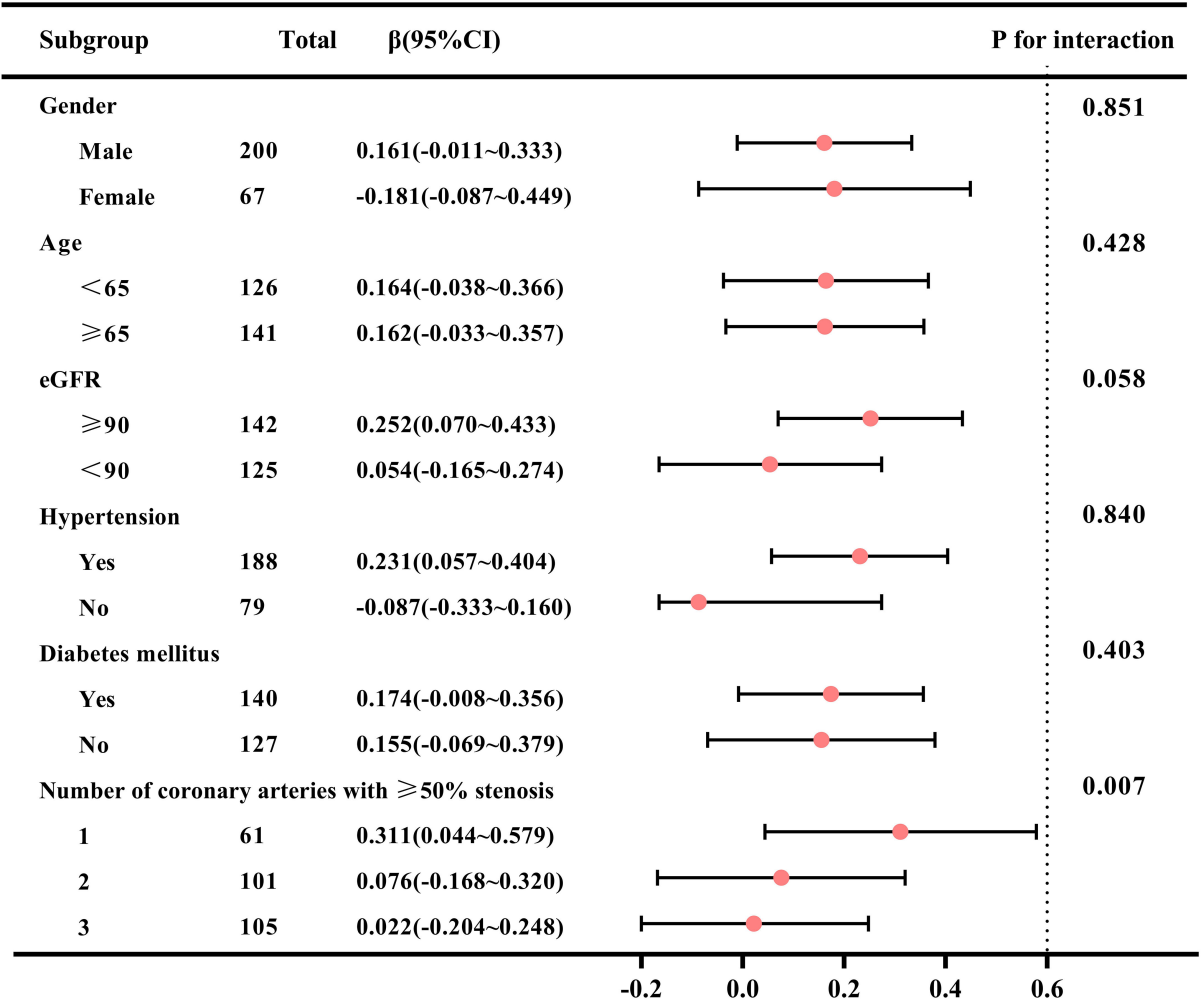
Subgroup analysis of correlation between TyG index and GWW in patients with CAD. Note: GWW was converted by natural logarithmic function. TyG, triglyceride–glucose; GWW, global wasteful work; CAD, coronary artery disease; eGFR, estimated glomerular filtration rate.

The forest plot of the subgroup analysis is shown in **Figure 5**. Subgroup analysis showed a significant interaction between the number of coronary arteries with ≥50% stenosis and the TyG index (P for interaction=0.007). The association between the TyG index and GWW was more significant in single-vessel lesion than in other subgroups (β=0.311; 95% CI 0.044~0.579 for single-vessel lesion vs. β=0.076; 95% CI -0.168~0.320 for two-vessel lesions vs. β=0.022; 95% CI -0.204~0.248 for three-vessel lesions). The association between the TyG index and GWW was similar in subgroups of patients stratified by age, sex, eGFR, hypertension and diabetes mellitus (P values for interaction>0.05).

## Discussion

In this study, we observed that TyG index was positively associated with GWW in patients with CAD. This correlation existed after adjusting for the effects of confounding factors such as age, gender, smoking, drinking, BMI, SBP, HbA1c, TC and LDL-c, indicating that elevated TyG index level may be independently correlated with early subclinical myocardial dysfunction.

The TyG index, derived from FBG and TG, was proven to be closely related with insulin resistance and metabolic syndrome. Previous studies have demonstrated that TyG index might be a valuable predictor on cardiac function in different populations. The ARIC study enrolled participants free of history of CAD and HF at baseline, and showed that an elevated TyG index was significantly associated with a higher risk of incident HF and impaired left ventricular structure and function (19). Ye et al. (20) demonstrated that TyG index was independently associated with left ventricular diastolic function in asymptomatic individuals from southwest China, and might serve as an effective tool to identify left ventricular diastolic dysfunction during routine health check-ups in primary care. A study by Wang et al. (21) suggested that in diabetic patients, TyG index was positively correlated with greater risk of developing HFpEF. In another study, Chen et al. (22) for the first time explored the relationship between TyG index and left ventricular function in T2DM patients with preserved ejection fraction, and it was found that the increased TyG index was associated with decreased GLS, which illustrated the TyG index could be adopted as a sensitive and practical index to predict subclinical left ventricular systolic dysfunction. However, in patients with CAD, few researches on the correlation between TyG index and early subclinical myocardial dysfunction have been published. Recently, Lin et al. (1) conducted a cross-sectional study to investigate the association between TyG index and myocardial remodeling during subclinical stage in CAD patients, and also indicated that TyG index was negatively correlated with GLS, especially in female. This current study confirmed that there was a correlation between TyG index and GLS, but it was significantly weakened after adjusting for confounding factors (**Supplementary Tables S1**, **S2**). It might be due to the afterload dependence of GLS, therefore, we used MW measurements, which were more suitable as indicators of cardiac function in subclinical stage, to conduct the study of association with TyG index.

Our study indicated that, among the four MW parameters, only GWW increased with the rise of TyG index, while GCW, GWI and GWE did not exhibit reliable or stable correlations with TyG index. To our knowledge, this has not been reported previously. GWW quantifies the energy consumed by the myocardium that is wasted and does not contribute to cardiac output. This research focused on the reduction of myocardial ineffective energy consumption, which could help prevent myocardial impairment in CAD patients during the subclinical stage of cardiac dysfunction.

Analysis of healthy individuals derived from STAAB cohort study showed that GCW and GWI correlated with left ventricular systolic function (LVEF, GLS), while diastolic function (E/e’, left atrium volume) was correlated with GWW (23). Therefore, it might infer that GWW is one of the first parameters to change in the occurrence of early subclinical myocardial damage. Liao et al. (24) enrolled T2DM patients without clinical cardiovascular diseases to explore the role of MW measurements. Compared with healthy controls, they found that GWW was significantly increased, GWI and GWE were significantly decreased in T2DM patients with no difference in GCW. Another study from Cao et al. (25) had similar results, except that GWI was insignificantly different between two groups. They also came up with a particular result that an elevated peak strain dispersion (PSD) was found in diabetics and associated positively with GWW. PSD represents the standard deviation of the time-to-peak longitudinal strain for each left ventricular segment over the entire cardiac cycle, which is used to evaluate the synchronicity of left ventricular myocardial deformation (26). The uncoordinated left ventricular myocardial strain might be one of the causes of the elevated GWW.

It has been widely discussed that the underlying mechanisms of the correlation between increased TyG index and early subclinical myocardial dysfunction in CAD patients. The alteration of glucose and lipid metabolisms are possibly responsible for cardiac structural and functional adaptations (27). Prolonged hyperglycemia can induce glycosylation reaction between proteins and glucose to accumulate advanced glycation end products (AGEs). Binding of AGEs to their receptors (RAGEs) activates multiple intracellular signaling pathways, stimulating fibroblast differentiate into myofibroblast, increasing extracellular matrix accumulation, then promoting myocardial fibrosis (28, 29). The reduced glucose uptake as a result of insulin resistance facilitates increased mitochondrial fatty acid uptake in cardiomyocytes and induce an accumulation of toxic lipid metabolites, resulting in cardiac lipotoxicity and mitochondrial dysfunction, stimulating ROS production and oxidative stress (30–32). On the other hand, Chronic high glucose and insulin concentrations also exert deleterious effects on endoplasmic reticulum and induce abnormalities of calcium handling (30, 31, 33). The interactions of these alterations jointly promote myocardial fibrosis and diastolic dysfunction. Metabolic disorders can activate renin-angiotensin-aldosterone system (RAAS), increase angiotensin II and aldosterone activity, which leads to cardiomyocyte hypertrophy, increased cardiac fibroblast proliferation and myocardial remodeling acceleration (32, 34). Activation of the RAAS may induce insulin resistance through the mTOR-S6K1 signal transduction pathway (35, 36).

### Strength and limitations

To the best of our knowledge, this is the first study to explore the TyG index and MW parameters in CAD patients. However, the study still had some limitations. First, because of the inherent disadvantages of cross-sectional studies, we cannot infer a causal relationship during this study. Second, as a single-center study with a small sample size, the population characteristics were relatively simple, which may be the reason why no significant differences were found in traditional subgroup analyses. Subgroup analysis showed that the correlation between the TyG index and GWW was not significant in the population with multi-vessel disease, which may be due to factors such as collateral circulation and varying degrees of stenosis in different branches. Therefore, it will be necessary to increase the sample size for different types of lesions in future studies. Third, PSL technology is based on two-dimensional speckle tracking imaging, which requires a high-quality ultrasound image. As a result, some patients upon speckle-tracking analysis failure had to be excluded, which could lead to the risk of reporting bias. Fourth, since the severity of coronary artery occlusion as well as angina symptoms could impact MW, the difference in MW before and after PCI could provide insights into the effects of revascularization on myocardial efficiency, which needs further study in the future.

### Conclusions

Our results demonstrated that an elevated TyG index was independently correlated with early subclinical myocardial dysfunction in CAD patients. The strict control of TyG index may be conducive to reduce the meaningless myocardial energy consumption, so as to forestall the progression of clinical HF in CAD patients.

## Data Availability

The data analyzed in this study is subject to the following licenses/restrictions: The datasets generated and analyzed during the current study are not publicly available due privacy and ethical restrictions but are available from the corresponding author on reasonable request. Requests to access these datasets should be directed to bjh_wangfang@163.com.

## References

[1] Na L Cui W Li X Chang J Xue X. Association between the triglyceride-glucose index and left ventricular global longitudinal strain in patients with coronary heart disease in Jilin Province, China: a cross-sectional study. Cardiovasc Diabetol. (2023) 22:321. doi: 10.1186/s12933-023-02050-9 37993858 PMC10666388

[2] Han D Kim SH Shin DG Kang MK Choi S Lee N. Prognostic implication of platelet reactivity according to left ventricular systolic dysfunction status in patients treated with drug-eluting stent implantation: analysis of the PTRG-DES consortium. J Korean Med Sci. (2024) 39:e27. doi: 10.3346/jkms.2024.39.e27 38258362 PMC10803212

[3] Cauwenberghs N Knez J Thijs L Haddad F Vanassche T Yang WY. Relation of insulin resistance to longitudinal changes in left ventricular structure and function in a general population. J Am Heart Assoc. (2018) 7:e008315. doi: 10.1161/JAHA.117.008315 29574459 PMC5907600

[4] Frişan AC Mornoş C Lazăr MA Şoşdean R Crişan S Ionac I. Echocardiographic myocardial work: A novel method to assess left ventricular function in patients with coronary artery disease and diabetes mellitus. Medicina (Kaunas). (2024) 60:199. doi: 10.3390/medicina60020199 38399487 PMC10890444

[5] Banerjee D Biggs ML Mercer L Mukamal K Kaplan R Barzilay J. Insulin resistance and risk of incident heart failure: Cardiovascular Health Study. Circ Heart Fail. (2013) 6:364–70. doi: 10.1161/CIRCHEARTFAILURE.112.000022 PMC3888807 23575256

[6] Khalaji A Behnoush AH Khanmohammadi S Ghanbari Mardasi K Sharifkashani S Sahebkar A. Triglyceride-glucose index and heart failure: a systematic review and meta-analysis. Cardiovasc Diabetol. (2023) 22:244. doi: 10.1186/s12933-023-01973-7 37679763 PMC10486123

[7] Dong S Zhao Z Huang X Ma M Yang Z Fan C. Triglyceride-glucose index is associated with poor prognosis in acute coronary syndrome patients with prior coronary artery bypass grafting undergoing percutaneous coronary intervention. Cardiovasc Diabetol. (2023) 22:286. doi: 10.1186/s12933-023-02029-6 37891647 PMC10612342

[8] Alavi Tabatabaei G Mohammadifard N Rafiee H Nouri F Maghami Mehr A Najafian J. Association of the triglyceride glucose index with all-cause and cardiovascular mortality in a general population of Iranian adults. Cardiovasc Diabetol. (2024) 23:66. doi: 10.1186/s12933-024-02148-8 38347581 PMC10863153

[9] Sánchez-García A Rodríguez-Gutiérrez R Mancillas-Adame L González-Nava V Díaz González-Colmenero A Solis RC. Diagnostic accuracy of the triglyceride and glucose index for insulin resistance: A systematic review. Int J Endocrinol. (2020) 2020:4678526. doi: 10.1155/2020/4678526 32256572 PMC7085845

[10] Marwick TH. Ejection fraction pros and cons: JACC state-of-the-art review. J Am Coll Cardiol. (2018) 72:2360–79. doi: 10.1016/j.jacc.2018.08.2162 30384893

[11] Chan J Shiino K Obonyo NG Hanna J Chamberlain R Small A. Left ventricular global strain analysis by two-dimensional speckle-tracking echocardiography: the learning curve. J Am Soc Echocardiogr. (2017) 30:1081–90. doi: 10.1016/j.echo.2017.06.010 28797723

[12] Kalam K Otahal P Marwick TH. Prognostic implications of global LV dysfunction: a systematic review and meta-analysis of global longitudinal strain and ejection fraction. Heart. (2014) 100:1673–80. doi: 10.1136/heartjnl-2014-305538 24860005

[13] Marzlin N Hays AG Peters M Kaminski A Roemer S O’Leary P. Myocardial work in echocardiography. Circ Cardiovasc Imaging. (2023) 16:e01441. doi: 10.1161/CIRCIMAGING.122.014419 36734221

[14] Russell K Eriksen M Aaberge L Wilhelmsen N Skulstad H Remme EW. A novel clinical method for quantification of regional left ventricular pressure-strain loop area: a non-invasive index of myocardial work. Eur Heart J. (2012) 33:724–33. doi: 10.1093/eurheartj/ehs016 PMC3303715 22315346

[15] Levey AS Stevens LA Schmid CH Zhang YL Castro AF 3rd Feldman HI. CKD-EPI (Chronic Kidney Disease Epidemiology Collaboration). A new equation to estimate glomerular filtration rate. Ann Intern Med. (2009) 150:604–12. doi: 10.7326/0003-4819-150-9-200905050-00006 PMC2763564 19414839

[16] Simental-Mendía LE Rodríguez-Morán M Guerrero-Romero F. The product of fasting glucose and triglycerides as surrogate for identifying insulin resistance in apparently healthy subjects. Metab Syndr Relat Disord. (2008) 6:299–304. doi: 10.1089/met.2008.0034 19067533

[17] American Diabetes Association. Diagnosis and classification of diabetes mellitus. Diabetes Care. (2013) 36 Suppl 1:S67–74. doi: 10.2337/dc13-S067 PMC3537273 23264425

[18] Gensini GG. A more meaningful scoring system for determining the severity of coronary heart disease. Am J Cardiol. (1983) 51:606. doi: 10.1016/s0002-9149(83)80105-2 6823874

[19] Huang R Lin Y Ye X Zhong X Xie P Li M. Triglyceride-glucose index in the development of heart failure and left ventricular dysfunction: analysis of the ARIC study. Eur J Prev Cardiol. (2022) 29:1531–41. doi: 10.1093/eurjpc/zwac058 35512245

[20] Ye R Zhang X Zhang Z Wang S Liu L Jia S. Association of cardiometabolic and triglyceride-glucose index with left ventricular diastolic function in asymptomatic individuals. Nutr Metab Cardiovasc Dis. (2024) 34(7):1590–600. doi: 10.1016/j.numecd.2024.02.008 38499451

[21] Wang T Xu J Zhang H Tao L Huang X. Triglyceride-glucose index for the detection of subclinical heart failure with preserved ejection fraction in patients with type 2 diabetes. Front Cardiovasc Med. (2023) 10:1086978. doi: 10.3389/fcvm.2023.1086978 36793475 PMC9923050

[22] Chen Y Fu J Wang Y Zhang Y Shi M Wang C. Association between triglyceride glucose index and subclinical left ventricular systolic dysfunction in patients with type 2 diabetes. Lipids Health Dis. (2023) 22:35. doi: 10.1186/s12944-023-01796-1 36890516 PMC9993628

[23] Morbach C Sahiti F Tiffe T Cejka V Eichner FA Gelbrich G. Myocardial work - correlation patterns and reference values from the population-based STAAB cohort study. PloS One. (2020) 15:e0239684. doi: 10.1371/journal.pone.0239684 33031416 PMC7544116

[24] Liao L Shi B Ding Z Chen L Dong F Li J. Echocardiographic study of myocardial work in patients with type 2 diabetes mellitus. BMC Cardiovasc Disord. (2022) 22:59. doi: 10.1186/s12872-022-02482-3 35172745 PMC8851829

[25] Cao W Deng Y Lv L Liu X Luo A Yin L. Assessment of left ventricular function in patients with type 2 diabetes mellitus by non-invasive myocardial work. Front Endocrinol (Lausanne). (2023) 5:1241307. doi: 10.3389/fendo.2023.1241307 PMC10508289 37732124

[26] Minczykowski A Guzik P Sajkowska A Pałasz-Borkowska A Wykrętowicz A. Interrelationships between peak strain dispersion, myocardial work indices, isovolumetric relaxation and systolic-diastolic coupling in middle-aged healthy subjects. J Clin Med. (2023) 12:5623. doi: 10.3390/jcm12175623 37685690 PMC10488442

[27] Evangelista I Nuti R Picchioni T Dotta F Palazzuoli A. Molecular dysfunction and phenotypic derangement in diabetic cardiomyopathy. Int J Mol Sci. (2019) 20:3264. doi: 10.3390/ijms20133264 31269778 PMC6651260

[28] Zeng Y Li Y Jiang W Hou N. Molecular mechanisms of metabolic dysregulation in diabetic cardiomyopathy. Front Cardiovasc Med. (2024) 11:1375400. doi: 10.3389/fcvm.2024.1375400 38596692 PMC11003275

[29] Khalid M Petroianu G Adem A. Advanced glycation end products and diabetes mellitus: mechanisms and perspectives. Biomolecules. (2022) 12:542. doi: 10.3390/biom12040542 35454131 PMC9030615

[30] Jia G Whaley-Connell A Sowers JR. Diabetic cardiomyopathy: a hyperglycaemia- and insulin-resistance-induced heart disease. Diabetologia. (2018) 61:21–8. doi: 10.1007/s00125-017-4390-4 PMC5720913 28776083

[31] Jia G DeMarco VG Sowers JR. Insulin resistance and hyperinsulinaemia in diabetic cardiomyopathy. Nat Rev Endocrinol. (2016) 12:144–53. doi: 10.1038/nrendo.2015.216 PMC4753054 26678809

[32] Prandi FR Evangelista I Sergi D Palazzuoli A Romeo F. Mechanisms of cardiac dysfunction in diabetic cardiomyopathy: molecular abnormalities and phenotypical variants. Heart Fail Rev. (2023) 28:597–606. doi: 10.1007/s10741-021-10200-y 35001338

[33] Marciniak SJ Ron D. Endoplasmic reticulum stress signaling in disease. Physiol Rev. (2006) 86:1133–49. doi: 10.1152/physrev.00015.2006 17015486

[34] Jeong S Lee JH. The verification of the reliability of a triglyceride-glucose index and its availability as an advanced tool. Metabolomics. (2021) 17:97. doi: 10.1007/s11306-021-01837-9 34724122

[35] Kim JA Jang HJ Martinez-Lemus LA Sowers JR. Activation of mTOR/p70S6 kinase by ANG II inhibits insulin-stimulated endothelial nitric oxide synthase and vasodilation. Am J Physiol Endocrinol Metab. (2012) 302:E201–8. doi: 10.1152/ajpendo.00497.2011 PMC3340897 22028412

[36] Jia G Aroor AR Martinez-Lemus LA Sowers JR. Overnutrition, mTOR signaling, and cardiovascular diseases. Am J Physiol Regul Integr Comp Physiol. (2014) 307:R1198–206. doi: 10.1152/ajpregu.00262.2014 PMC4233289 25253086

