# Supplementary material for: Association between the triglyceride–glucose index and left ventricular myocardial work indices in patients with coronary artery disease
Source: Front Endocrinol (Lausanne). 2024 Oct 24;15:1447984. doi: 10.3389/fendo.2024.1447984 (PMC11544542; doi:10.3389/fendo.2024.1447984)
Supplement: Supplementary file 1 [file Table1.docx]

**Supplementary Table 1. multivariate analysis of parameters associated between TyG index and GLS**

| Model | β | 95% CI | *P* value |
| --- | --- | --- | --- |
| Model 1 | -0.193 | -1.302 to -0.311 | 0.002 |
| Model 2 | -0.722 | -1.236 to -0.208 | 0.006 |
| Model 3 | -0.531 | -1.137 to 0.076 | 0.086 |

Model 1: unadjusted.

Model 2: adjusted for age, sex, smoking and drinking.

Model 3: adjusted for Model 2 covariates + SBP, BMI, HbA1c, TC and LDL-C.

TyG, triglyceride–glucose; GLS, global longitudinal strain; CI, confidence interval; SBP, systolic blood pressure; BMI, body mass index; HbA1c, glycated hemoglobin A1c; TC, total cholesterol; LDL-C, low-density lipoprotein cholesterol.

**Supplementary Table 2. Trend test of changes in TyG index and GLS**

| Model | Quartile of TyG index | | | | P for trend |
| --- | --- | --- | --- | --- | --- |
|  | Q1(＜6.70)  β (95% CI) | Q2(6.70-7.09)  β (95% CI) | Q3(7.09-7.53)  β (95% CI) | Q4(＞7.53)  β (95% CI) |  |
| Model 1 | Reference | 0.773  (-1.139~0.624) | 0.512  (-1.544~0.205) | 0.234  (-2.329~-0.573) | 0.001 |
| Model 2 | Reference | 0.761  (-1.155~0.610) | 0.515  (-1.539~0.212) | 0.262  (-2.247~-0.429) | 0.003 |
| Model 3 | Reference | 1.242  (-0.684~1.119) | 0.763  (-1.162~0.621) | 0.353  (-2.069~-0.013) | 0.036 |

Model 1: unadjusted.

Model 2: adjusted for age, sex, smoking and drinking.

Model 3: adjusted for Model 2 covariates + SBP, BMI, HbA1c, TC and LDL-C.

TyG, triglyceride–glucose; GLS, global longitudinal strain; CI, confidence interval; SBP, systolic blood pressure; BMI, body mass index; HbA1c, glycated hemoglobin A1c; TC, total cholesterol; LDL-C, low-density lipoprotein cholesterol.

**Supplementary Table 3. multivariate analysis of parameters associated between TyG index and GCW**

| Model | β | 95% CI | *P* value |
| --- | --- | --- | --- |
| Model 1 | -44.422 | -126.176 to 37.333 | 0.286 |
| Model 2 | -24.830 | -109.265 to 59.604 | 0.563 |
| Model 3 | -37.105 | -121.677 to 47.467 | 0.388 |

Model 1: unadjusted.

Model 2: adjusted for age, sex, smoking and drinking.

Model 3: adjusted for Model 2 covariates + SBP, BMI, HbA1c, TC and LDL-C.

TyG, triglyceride–glucose; GCW, global useful work; CI, confidence interval; SBP, systolic blood pressure; BMI, body mass index; HbA1c, glycated hemoglobin A1c; TC, total cholesterol; LDL-C, low-density lipoprotein cholesterol.

**Supplementary Table 4. multivariate analysis of parameters associated between TyG index and GWI**

| Model | β | 95% CI | *P* value |
| --- | --- | --- | --- |
| Model 1 | -63.272 | -136.104 to 9.561 | 0.088 |
| Model 2 | -50.848 | -126.770 to 25.074 | 0.188 |
| Model 3 | -83.068 | -161.354 to -4.783 | 0.038 |

Model 1: unadjusted.

Model 2: adjusted for age, sex, smoking and drinking.

Model 3: adjusted for Model 2 covariates + SBP, BMI, HbA1c, TC and LDL-C.

TyG, triglyceride–glucose; GWI, global work index; CI, confidence interval; SBP, systolic blood pressure; BMI, body mass index; HbA1c, glycated hemoglobin A1c; TC, total cholesterol; LDL-C, low-density lipoprotein cholesterol.

**Supplementary Table 5. multivariate analysis of parameters associated between TyG index and GWE**

| Model | β | 95% CI | *P* value |
| --- | --- | --- | --- |
| Model 1 | 0.540 | 0.139 to 0.942 | 0.008 |
| Model 2 | 0.495 | 0.067 to 0.923 | 0.024 |
| Model 3 | 0.479 | -0.037 to 0.995 | 0.069 |

Model 1: unadjusted.

Model 2: adjusted for age, sex, smoking and drinking.

Model 3: adjusted for Model 2 covariates + SBP, BMI, HbA1c, TC and LDL-C.

TyG, triglyceride–glucose; GWE, global work efficiency; CI, confidence interval; SBP, systolic blood pressure; BMI, body mass index; HbA1c, glycated hemoglobin A1c; TC, total cholesterol; LDL-C, low-density lipoprotein cholesterol.
